# Improved sensitivity roll-off in dual reference, buffered spectral-domain optical coherence tomography

**DOI:** 10.1117/1.JBO.26.2.025001

**Published:** 2021-02-10

**Authors:** David O. Otuya, Yogesh Verma, Romain Luu, Hamid Farrrokhi, Guillermo J. Tearney

**Affiliations:** a Massachusetts General Hospital, Harvard Medical School and the Wellman Center for Photomedicine, Boston, Massachusetts, United States; b Harvard-MIT Division of Health Sciences and Technology, Cambridge, Massachusetts, United States; c Harvard Medical School and Massachusetts General Hospital, Department of Pathology, Boston, Massachusetts, United States

**Keywords:** optical coherence tomography, *in vivo*, gastrointestinal tract, tethered capsule, endomicroscopy

## Abstract

**Significance:** While spectral-domain optical coherence tomography (SD-OCT) is a preferred form of OCT imaging, sensitivity roll-off limits its applicability for certain biomedical imaging applications.

**Aim:** The aim of this work is to extend the imaging range of conventional SD-OCT systems for imaging large luminal organs such as the gastrointestinal tract.

**Approach:** We present an SD-OCT system operating at a center wavelength of 1300 nm that uses two delayed reference arms to reduce sensitivity roll-off and an optical switch and a fiber optic delay line to ensure that the interference spectra are acquired from the same sample time window.

**Result:** The proposed system was used to image swine colon *ex vivo* and duodenum *in vivo*, demonstrating improved image quality due to a ∼14  dB increase in sensitivity at the edges of the ranging depth.

**Conclusion:** The proposed system requires modest hardware implementation and is compatible with catheter-based endoscopic helical scanning with enhanced sensitivity for the samples at a distance of ∼6  mm from the zero delay point.

## 1 Introduction

Spectral-domain optical coherence tomography (SD-OCT) offers positive attributes of high speed, high sensitivity, and phase stability, making it an attractive form of OCT imaging.^1^^–^^3^ As opposed to swept-source OCT (SS-OCT), it furthermore can be implemented over the entire visible and near-infrared (NIR), owing to the availability of spatially coherent broad bandwidth sources across this portion of the optical electromagnetic spectrum.^4^ However, SD-OCT suffers from a depth-dependent sensitivity decay that is undesirable for imaging tissues with irregular surface topology and large luminal organs where the distance between the probe and the tissue surface often varies by a large amount.^5^^,^^6^ In SD-OCT, the sensitivity roll-off is mainly determined by the spectral resolution of the spectrometer, which is governed by the spectrometer optics and the finite size and number of CCD pixels in its linear detection array.^7^ Another issue with SD-OCT is that the Fourier transform of the real interference signal leads to wrapped images on both sides of the zero-delay, and hence one cannot readily distinguish between negative and positive image depths.^2^^,^^3^^,^^8^^,^^9^ To avoid this ambiguity, the zero delay is usually set at or outside the tissue’s surface, leading to a further reduction in sensitivity at the end of the ranging depth.

Various efforts have been directed toward decreasing the sensitivity roll-off of SD-OCT. Several schemes incorporate reference or sample arm phase shifting to introduce a carrier frequency that enables quadrature detection.^10^^–^^12^ When this complex spectral interference signal is Fourier transformed,^10^^–^^12^ the sensitivity roll-off at the deep edge of the scan can be diminished by placing the zero delay in the middle of the ranging depth.^10^^–^^12^ The detection of such quadrature signals has also been explored using multi-arm fiber couplers (FCs)^13^^–^^15^ and polarization-sensitive measurement configurations.^16^

In addition to quadrature detection approaches, the use of dual reference arms to decrease sensitivity roll-off has been explored. One such scheme, by Wang et al.,^17^ uses an optical switch (OS) in the interferometer’s reference arm to alternate between two different reference arm delays. Interference spectra corresponding to each reference delay are sequentially acquired using a single spectrometer. Images are combined by concatenating the most sensitive part of the two cropped images that corresponds to either of the two reference arms with the better sensitivity. While this approach recovers sensitivity at increased depths, aliasing artifacts in the second image needed to be resolved by introducing a phase shift through decentering the optical beam from the pivot of a scanning galvanometer mirror.^18^ This feature makes it difficult to employ this approach for *in vivo* endoscopic OCT imaging that requires helical catheter scanning. Zotter et.al.^12^ demonstrated a dual reference arm approach for simultaneous acquisition of SD-OCT interference signals generated by two distinct reference arms, by use of two separate interferometers with separate light sources and line scan cameras. While the maximum line rate of the spectrometers is maintained, this method is more complicated, bulky, and costly than single spectrometer designs. Recently, SD-OCT employing an optical frequency comb was demonstrated to reduce sensitivity roll-off.^7^ Here, the multiplexing of multiple sequentially acquired spectra with slightly offset frequency combs was used to improve the sensitivity roll-off.^7^ A significant ∼14  dB improvement in the sensitivity roll-off was reported at the cost of the need for increased measurement time required for obtaining multiple interference spectra (M=6) for every A-line and the optical power loss from the Fabry–Perot interferometer that generated the optical frequency combs.

In this paper, we report a new SD-OCT configuration that uses two interferometers with different reference arm lengths. The output port of one of the interferometers is input into an optical fiber delay line (FDL) that delays the light by the A-line acquisition time.^19^ Both output ports are then input into an OS that alternates detection of light from each interferometer. In so doing, this SD-OCT configuration is able to obtain imaging of two distinct depth ranges while acquiring data from the same location on the sample. The resultant images are combined using an algorithm that stitches the two together, regaining ∼14  dB of sensitivity at the edges of the depth range while avoiding aliasing artifacts.

## 2 Methods

### 2.1 Dual Reference SDOCT System

**Fig. 1:**
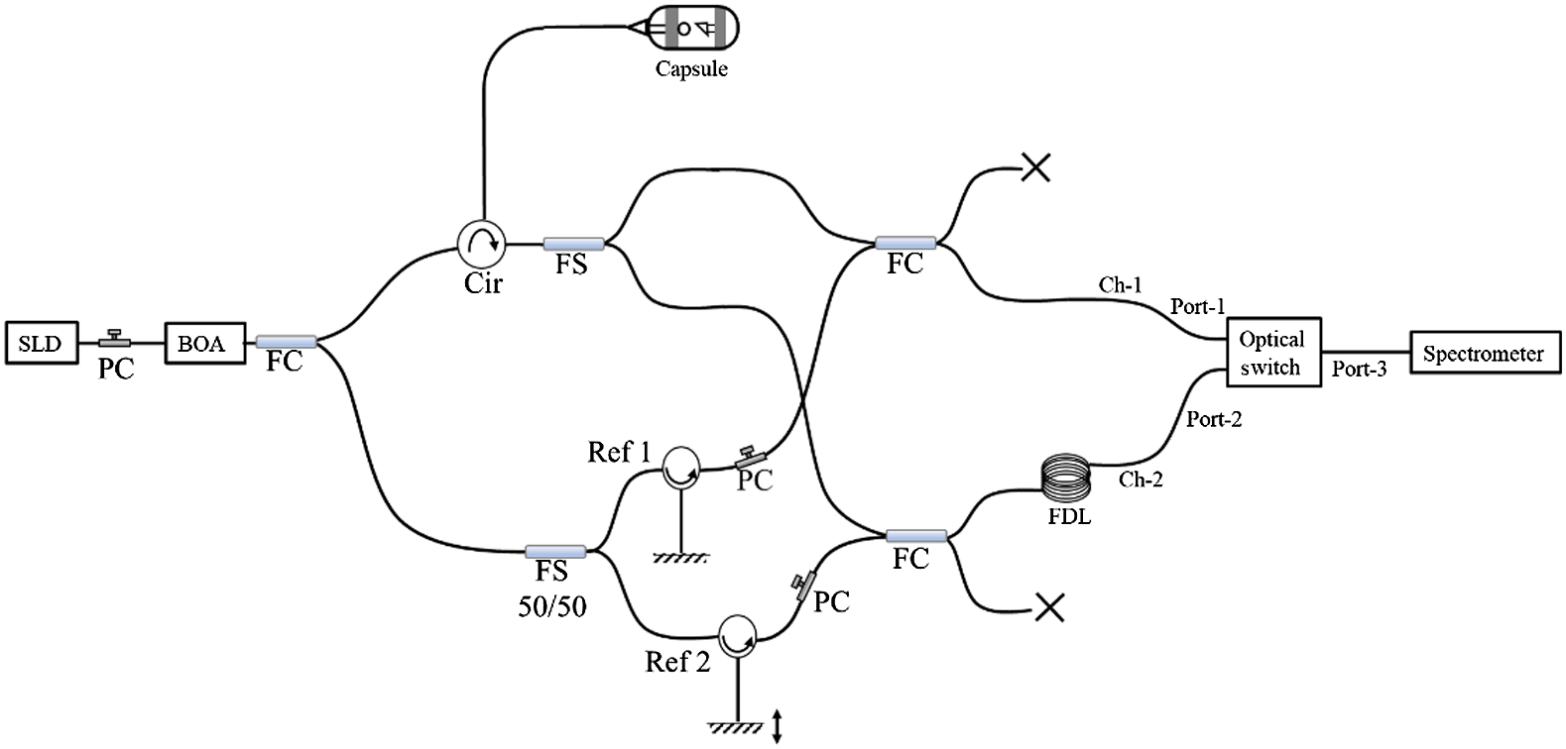
Schematic of the dual reference SD-OCT system. SLD: super luminescent diode, PC: polarization controller, BOA: booster optical amplifier, FC: fiber coupler, Cir: circulator, FS: fiber splitter, Ref. 1: reference arm 1, Ref. 2: reference arm 2, Ch-1: channel 1, and Ch-2: channel 2.

A schematic of the dual reference SD-OCT system is shown in Fig. 1. The configuration comprised a 3-mW superluminescent diode (SLD) with a 3-dB bandwidth of ∼100-nm centered at ∼1310  nm as a broadband light source and a booster optical amplifier (BOA) to amplify the broadband light to 22 mW. Since the light amplification by the BOA was polarization sensitive, a polarization controller (PC) was used between the SLD and BOA to optimize the light amplification and its spectral profile. A 75/25 coupler split the light from the BOA into the sample and reference arms. In the sample arm, a circulator was used to illuminate the sample and also to collect the back-reflected light from the sample. The back-scattered light from the sample was further split into two halves with a 50/50 fiber splitter (FS). In the reference arm, another 50/50 FS was used to split the light into the two reference arms. In one of the reference arms, a linear translation stage and collimation assembly were used to set a ∼3-mm predetermined optical path length delay between the two reference arms. 99/1 FCs were used to combine the light coming from the sample and reference arms such that 99% of the light coming from the sample was combined with 1% of the reference light. The 99% output from these couplers were passed to the spectrometer through an OS (AGILTRON 1310-nm NS SM 1×2 Switch NSSW-123121323, 500 kHz). While one of the 99/1 couplers was connected directly to the port 1 of 2×1 OS, the light output from the other 99/1 coupler was connected via a ∼1.5-km FDL (FDL to port 2 of the OS). Port 3 of the OS was connected to the spectrometer for detection of the interference spectra. The length of the FDL was chosen such that the temporal delay was equivalent to the single-line acquisition time of the line scan camera. Thus, delayed light from the FDL was available for detection every other acquisition cycle. This configuration ensured that successive back-scattered light from the same location of the sample within the same temporal window was detected.

The spectrometer (Wasatch Cobra Super SWIR 2K) used a 140-kHz InGaAs linear array (Sensors Unlimited GL2048R InGaAs Linescan Camera) to detect the spectral interferogram. The spectral resolution on the linear array was 0.075  nm/pixel, resulting in a theoretical 6-dB spectral roll-off of 2.5 mm. Two synchronized transistor-transistor logic (TTL) signals of frequencies of 140 and 70 kHz were generated using a dual-channel function generator to trigger the camera’s line acquisition and to drive the OS, respectively, such that the alternate lines acquired from camera corresponded to interference between the sample and the two different reference arms. The odd and even A-lines were deinterleaved and used to create two separate z-scans that corresponded to the two different reference arms. The data from the two A-lines were merged using the algorithm described in Sec. 2.2, resulting in an effective A-line rate of 70 kHz.

To demonstrate the performance of this SD-OCT system for imaging internal organs, an opto-mechanically engineered OCT imaging capsule known as a tethered capsule endomicroscopy (TCE) device^20^ with a distal scanning motor^21^ was used to image swine colon *ex vivo* and swine duodenum *in vivo*. The capsule had an outer diameter (OD) of 11 mm and a length of 25 mm; the 1-mm-diameter tether was 2-m long. The lateral resolution of the capsule’s optics was ∼30  μm. The capsule’s integrated motor^22^ rotated at 27 Hz. SD-OCT images comprised 2560 A-lines to capture the full circumference of the device and surrounding tissues.

### 2.2 Data Acquisition and Image Merging Algorithm

Acquired spectra were first separated corresponding to each reference arm and processed separately. The processing steps included background subtraction, resampling, fast Fourier transformation, and then mixing the images corresponding to the two reference arms with different optical delays while suppressing the aliasing artifacts to form a single OCT image.

**Fig. 2:**
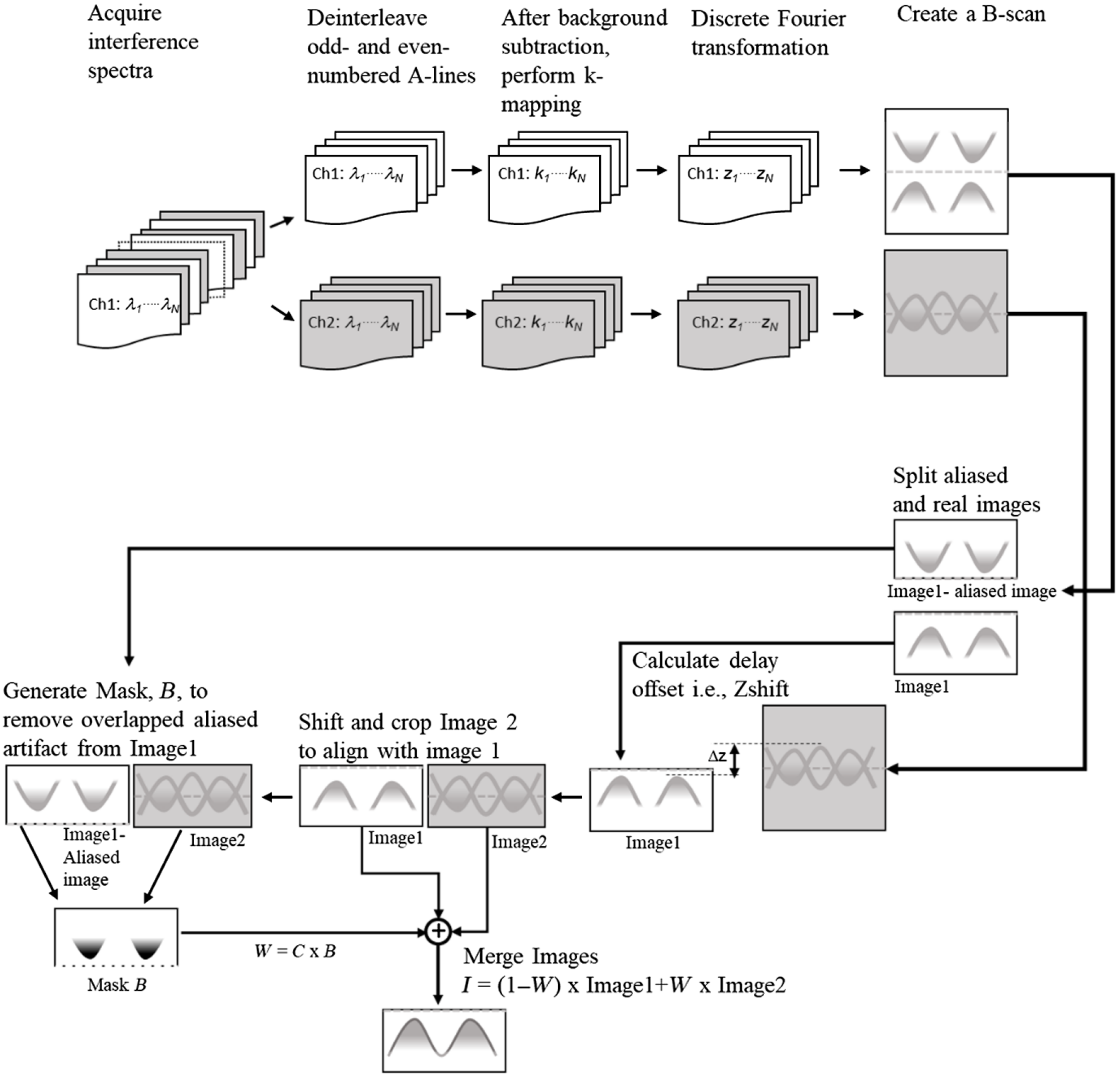
Schematic of the processing steps used to generate a combined image from images acquired from each interferometer.

Figure 2 is the flow chart diagram showing the steps undertaken to acquire and merge the two images corresponding to the two reference arms. Data acquisition was done such that M interference spectra containing N samples per spectrum were sampled at a uniform interval in the wavelength domain, resulting in a data matrix $I{\left(\lambda ,t\right)}_{M\times N}$ of M rows and N columns. Odd- and even-indexed rows of the spectra were separated, resulting in two data matrices $I_{ch1}{\left(\lambda ,t\right)}_{M/2\times N}$ and $I_{ch2}{\left(\lambda ,t\right)}_{M/2\times N}$, each with M/2 rows (spectra) and N columns (samples per spectrum). The background was then calculated from each data matrix by obtaining the mean of all the columns in the matrix. This operation was followed by background subtraction, where the mean vectors were subtracted from all the rows in the data matrices.

$$I_{ch1}^{\prime }{\left(\lambda ,t\right)}_{M/2\times N}=I_{ch1}{\left(\lambda ,t\right)}_{M/2\times N}-\text{mean}\{I_{ch1}{\left(\lambda ,t\right)}_{M/2\times N}\}\;I_{ch2}^{\prime }{\left(\lambda ,t\right)}_{M/2\times N}=I_{ch2}{\left(\lambda ,t\right)}_{M/2\times N}-\text{mean}\{I_{ch2}{\left(\lambda ,t\right)}_{M/2\times N}\}.$$ (1)

The linearly sampled data in wavelength domain (λ – domain) was then resampled linearly in the wavenumber domain (k-domain) using a linear interpolation remapping function.^23^

$$I_{ch1}^{\prime }{\left(\lambda ,t\right)}_{M/2\times N}\to I_{ch1}^{\prime }{\left(k,t\right)}_{M/2\times N}\;I_{ch2}^{\prime }{\left(\lambda ,t\right)}_{M/2\times N}\to I_{ch2}^{\prime }{\left(k,t\right)}_{M/2\times N}.$$ (2)

Fast Fourier transformation was performed on each interpolated array for the two data matrices to obtain the corresponding z-scans.

$$I_{ch1}{\left(z,t\right)}_{M/2\times N}=FFT(I_{ch1}^{\prime }{\left(k,t\right)}_{M/2\times N}),\;I_{ch2}{\left(z,t\right)}_{M/2\times N}=FFT(I_{ch2}^{\prime }{\left(k,t\right)}_{M/2\times N}).$$ (3)

The first z-scan $I_{ch1}{\left(z,t\right)}_{M/2\times N}$ was then split into two (unaliased and aliased) images such that the first half (from first column to N/2’th column) represented the aliased image $I_{ch1}^{\prime }{\left(\lambda ,t\right)}_{M/2\times N/2}$ of the second half (from column no N/2+1 to N). The spatial offset z-shift between the unaliased image from first channel $I_{ch1}{\left(z,t\right)}_{M/2\times N}$ and the image from second channel $I_{ch2}{\left(z,t\right)}_{M/2\times N}$ was then obtained by calculating their cross-correlation. Image $I_{ch2}{\left(z,t\right)}_{M/2\times N}$ was then shifted and cropped such that it was aligned and made to be equal in dimension to the first unaliased image $I_{ch1}{\left(z,t\right)}_{M/2\times N/2}$. The shifted and cropped image from the second channel $I_{ch2}{\left(z,t\right)}_{M/2\times N/2}$ was binarized using the image from channel 1 to generate a mask matrix $B_{M/2\times N}$. This mask was used to remove the overlapping aliased image in $I_{ch2}{\left(z,t\right)}_{M/2\times N/2}$. A weighted merging coefficient matrix $W_{M/2\times N/2}=C_{M/2\times N/2}\cdot B_{M/2\times N/2}$ was then calculated, where $C_{M/2\times N/2}$ was a weight function matrix. The weight function matrix was derived from a linear function f(z)=constant×z or a sigmoid function:

$$f(z)=\frac{e^{\frac{(2z-z_{n})\pi }{z_{n}}}}{\left(e^{\frac{(2z-z_{n})\pi }{z_{n}}}+1\right)},$$ (4)

where z denoted the imaging depth ranging from 0 to $z_{n}$ such that

$$C_{M/2\times N/2}=[\begin{array}{ccc} f(z_{1}) & \ldots & f(z_{n})\\ \vdots & \ldots & \vdots \\ f(z_{1}) & \ldots & f(z_{n}) \end{array}].$$ (5)

The two images $I_{ch1}{\left(z,t\right)}_{M/2\times N/2}$ and $I_{ch2}{\left(z,t\right)}_{M/2\times N/2}$ were then merged using the merging coefficient matrix to generate a final image $I_{m}{\left(z,t\right)}_{M/2\times N/2}$ using the following equation:

$$I_{m}{\left(z,t\right)}_{M/2\times N/2}=(1-W_{M/2\times N/2})\cdot I_{ch1}{\left(z,t\right)}_{M/2\times N/2}+W_{M/2\times N/2}\cdot I_{ch2}{\left(z,t\right)}_{M/2\times N/2}.$$ (6)

This merging algorithm was implemented in C++ for real-time data acquisition using a desktop computer (Dell Precision Tower 5810) running on eight threads. An offline Matlab implementation of the merging algorithm was also developed for data post-processing on a desktop computer (ASUS with Intel^®^ Core™ i7-4970k processor).

### 2.3 Swine Intestinal Imaging

A 20-cm-long piece of colon was resected from a 33 kg swine post-euthanasia (MGH IACUC protocol# 2016N000215). The TCE device was connected to the aforementioned SD-OCT system with an optical output of 22 mW. The capsule was inserted in the colon, immersed in phosphate-buffered saline, with pairs of hemostat scissors. The capsule was pulled back at a rate of ∼2  mm/s while the optics rotated at 27 fps, capturing helical scans of the colon wall.

The *in vivo* swine study was approved by the MGH IACUC committee (MGH IACUC protocol# 2016N000215). For this study, a 25-kg swine was sedated, anesthetized, and intubated prior to the imaging experiment. A gastroscope (Pentax EG-2990K) in an overtube (OD ∼15  mm, inner diameter ∼13  mm) was introduced into the duodenum. The gastroscope was retrieved leaving the overtube in place with its distal tip in the duodenum. The TCE capsule device was then introduced through the overtube to the duodenum. OCT imaging was conducted at 27 fps using the SD-OCT system for a duration of 5 min after which the capsule and the overtube were retrieved. An SS-OCT system consisting of an Axsun OCT engine (Axsun Technologies) with an A-line rate of 100 kHz connected to a TCE capsule running at 39 fps, acquiring the same number of A-lines per frame (2560 A-lines/frame) as the proposed SD-OCT system, was also used to acquire images *in vivo* in swine.

## 3 Results

### 3.1 Image Acquisition and Display

Data acquisition was performed at 140,000 A-lines/sec and displayed in real-time at 12 fps with the C++ implementation of the merging algorithm described in Sec. 2.2. The offline Matlab version of the merging scheme yielded a 27-fps frame rate. After optimization of the binarization threshold of the image from the second interferometer, the merging algorithm was robust and successful for all subsequent images.

### 3.2 Sensitivity Measurement

**Fig. 3:**
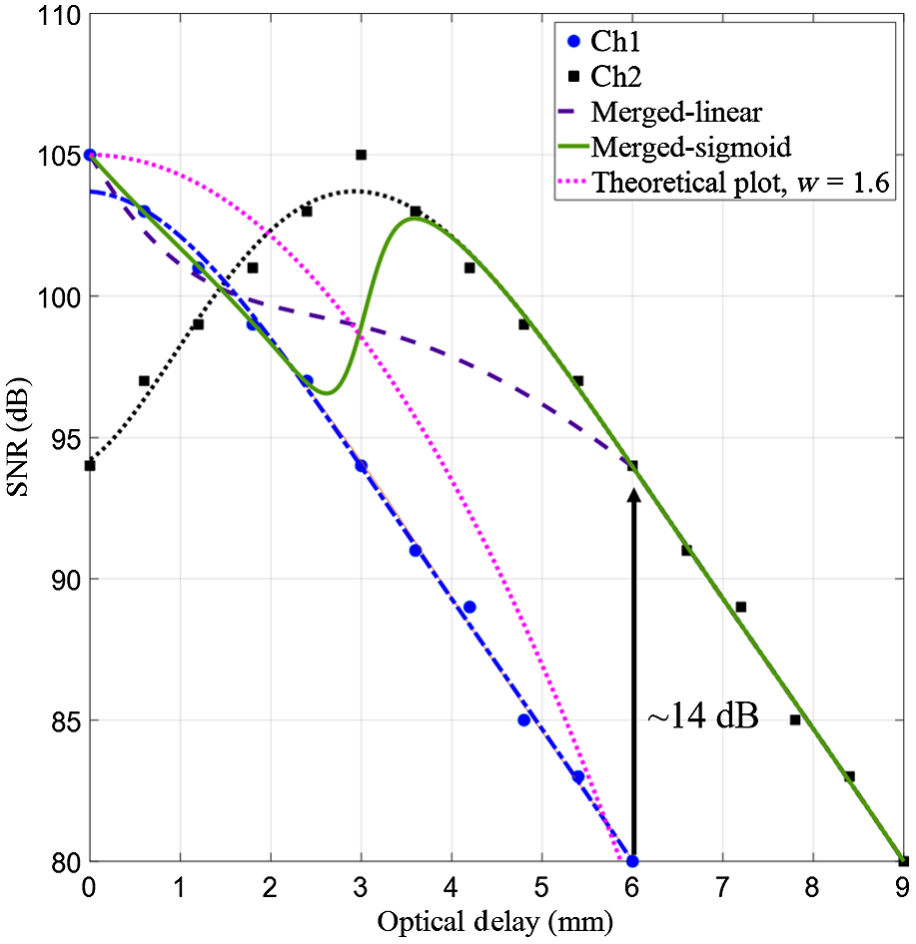
Measured sensitivity roll-off corresponding to the two reference arms. Blue circles show sensitivity measurement for interferometer 1, which had a zero delay near to the outer surface of the capsule and black squares show the sensitivity measurement for interferometer 2, which had a zero delay that was 3 mm away from the outer surface of the capsule. The purple dotted and green solid plots show the sensitivity of the merged signals when linear or sigmoid merging function were used, respectively. The dotted magenta plot shows a theoretical sensitivity roll-off plot with a w parameter of 1.6 according to Eq. (2) in Ref. 24.

**Fig. 4:**
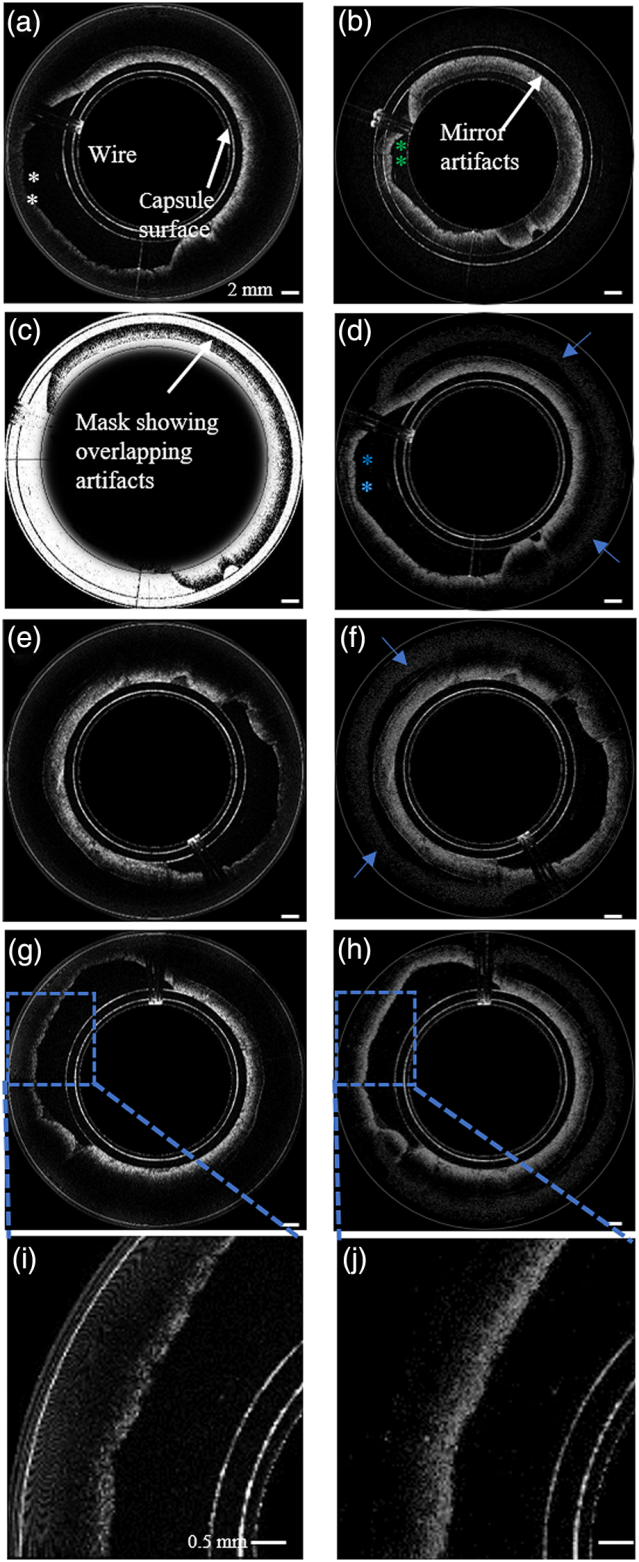
OCT images of swine colon *ex-vivo*. (a) OCT image corresponding to interferometer 1, with zero delay located at the outer surface of the capsule (Video 1); (b) OCT image corresponding to interferometer 2, with zero delay set to be 3 mm away from the outer surface of the capsule; (c) mask matrix computed to indicate areas where the aliased image overlapped the unaliased image in (b) corresponding to reference arm 2; (d) merged image showing increased imaging range (blue asterisks) achieved by combining images in (a) and (b) using the mask matrix in (c), (e), and (f); and (g) and (h) show corresponding image pairs obtained with single- and dual- interferometer SD-OCT systems, respectively (Video 2). (i) and (j) show the zoomed-in images of (g) and (h), respectively. The blue arrows in (d) and (f) indicate the artifacts created when the images are merged [Video 1, MP4, 29 MB] [URL: https://doi.org/10.1117/1.JBO.26.2.025001.1] [Video 2, MP4, 16 MB] [URL: https://doi.org/10.1117/1.JBO.26.2.025001.2].

The measured maximum sensitivity of our system corresponding to the interferometer 1 and interferometer 2 was 105 and 104 dB, respectively; the theoretically calculated sensitivity of the system was ∼107  dB. Figure 3 shows the sensitivity roll-off for the two interferometers. The blue and black plots represent the sensitivity values for the reference arms 1 and 2, respectively. The 6-dB roll-off for interferometers 1 and 2 were both 1.5 mm. The difference between the theoretical and measured roll-off results was likely due to inter-pixel cross-talk in the spectrometer.^25^ Using a 6-mm ranging depth (in air) that is typical for TCE imaging, the sensitivity decayed by 25 dB at the edge of the scan for interferometer 1. As expected, this sensitivity roll-off led to poor image quality for tissue located at larger optical delays [Fig. 4(a)]. With its reference arm 3-mm delayed with respect to that of interferometer 1, the sensitivity of the images produced by interferometer 2 was down by 11 dB at both the top and bottom of the depth range. Data acquired by interferometer 2 showed an expected higher image intensity at 3-mm imaging depth [Fig. 4(b)].

Extensive decreases in sensitivity at both ends of the depth range were mitigated when using the two interferometers. The zero delay of reference arm 1 was set at the outer surface of the capsule while the zero delay of the reference arm 2 was set to be ∼3  mm away from the outer surface of the capsule. The offset between the two reference arms was chosen such that for the 0 to 3 mm imaging window, the sensitivity (105 to 98 dB) was derived from interferometer 1 and for 3 to ∼6  mm, interferometer 2 contributed to sensitivity (105 to 98 dB). The dashed purple and solid green curves of Fig. 3 show the resultant sensitivity roll-off for linear and sigmoid merging functions, respectively. A sensitivity enhancement of about 14 dB was observed for the sample surfaces located within the 3 to 6 mm optical delay window.

### 3.3 Ex vivo Colon and in vivo Duodenal Imaging

**Fig. 5:**
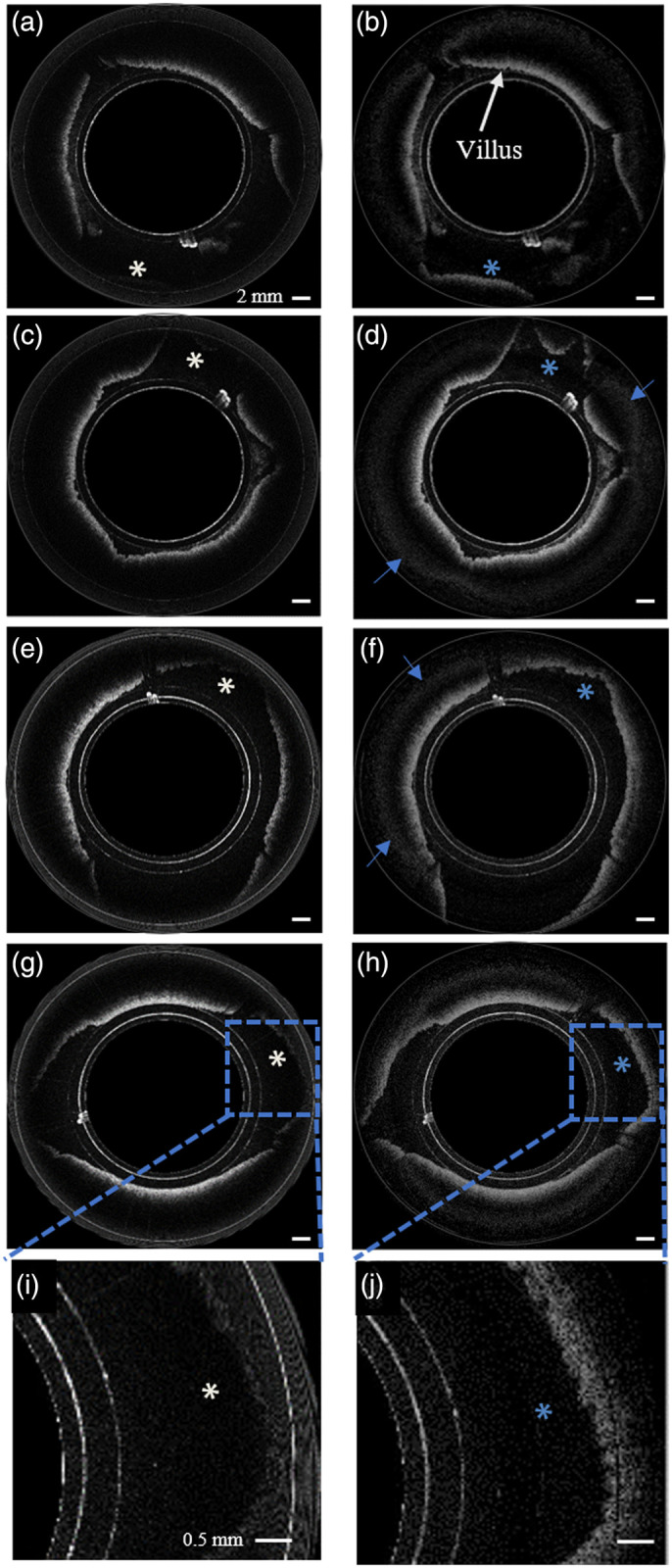
OCT images of swine duodenum *in vivo*. (a) OCT image with conventional SD-OCT system with diminished sensitivity at increased imaging depths shown by white asterisks; (b) image obtained with the newly developed SD-OCT system showing improved sensitivity at increased imaging range as shown by the blue asterisks, similar image pairs are shown in (c) and (d); (e) and (f); and (g) and (h). (i) and (j) show the zoomed-in images of (g) and (h), respectively. The blue arrows in (d) and (f) indicate the artifacts generated when the images were merged.

Figures 4(a) and 4(b) show images of a prosected colon acquired using the first and second interferometers, respectively. Setting the zero delay of the second interferometer at 3 mm caused tissue within the 0 to 3 mm window to be aliased into the real image as seen in Fig. 4(b). This inability to distinguish negative from positive image distances in OCT results from the complex conjugate ambiguity of the Fourier transform of real-valued spectrum obtained by the spectrometer. The mask matrix calculated according to the algorithm in Sec. 2.2 that shows areas of aliasing artifacts in Fig. 4(b) is shown in Fig. 4(c). The combined image formed by the merging algorithm described in Sec. 2 is shown in Fig. 4(d). As can be seen in the image acquired with a single reference arm [Fig. 4(a)] portions of the colon cross-section were poorly visible due to the loss of sensitivity at the periphery of the image. Our system and algorithm recovered data further from the device, allowing complete circumferential imaging of the colon wall [Fig. 4(d)]. Figure 5 shows *in vivo* images from four different locations of swine duodenum acquired by both single- and dual-interferometer SD-OCT systems. Figures 5(a), 5(c), 5(e), and 5(g) were acquired by the single interferometer system while the corresponding dual-interferometer images are shown in Figs. 5(b), 5(d), 5(f), and 5(h), respectively. As can be seen, there was a significant improvement in amount of bowel wall that can be visualized in cross section [Figs. 5(a) versus 5(b), 5(c) versus 5(d), 5(e) versus 5(f), and 5(g) versus 5(h)]. The blue arrows in Figs. 4(d), 4(f), 5(d), and 5(f) indicate artifacts, located deeper in the tissue away from surface, generated by merging images from the two channels.

**Fig. 6:**
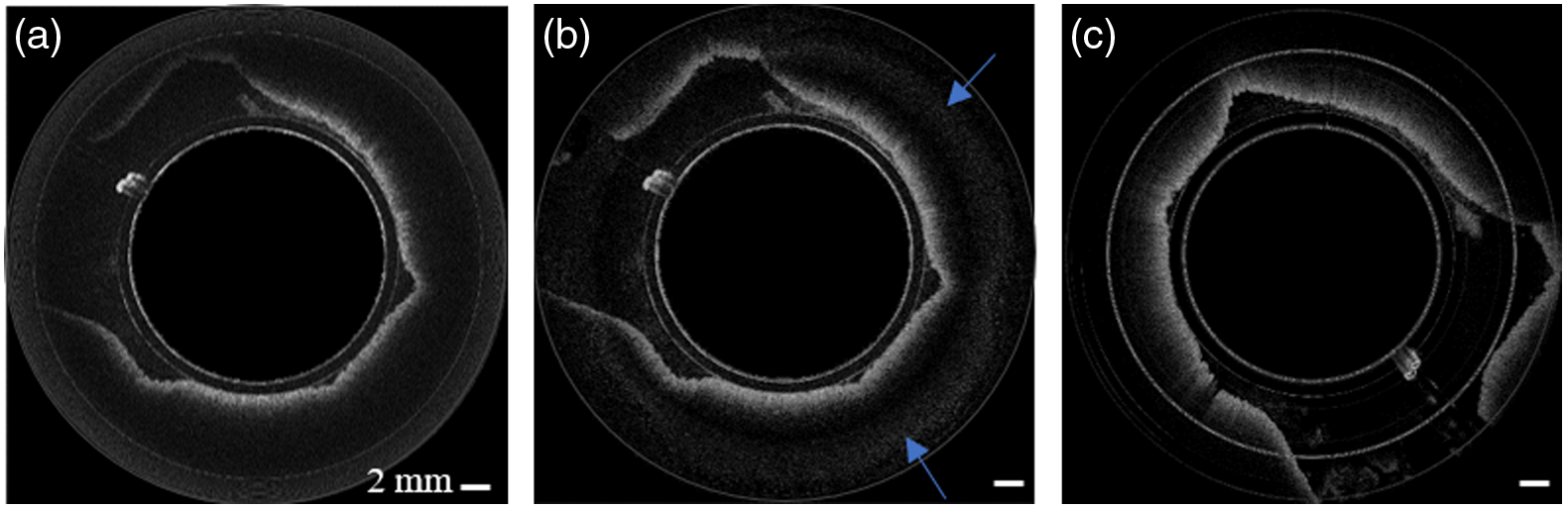
OCT images of swine duodenum *in vivo*. (a) using a conventional single-interferometer SD-OCT system; (b) when using the proposed dual-interferometer SD-OCT; and (c) OCT image obtained with a SS OCT system (Axsun Technologies). The blue arrows shown in (b) indicate artifacts deep the tissue generated from merging the two images.

Figures 6(a) and 6(b) show images acquired with the conventional single-interferometer and the dual-interferometer SD-OCT systems, respectively. Figure 6(c) shows an image acquired with the SS OCT system. As shown in Figs. 6(b) and 6(c), our SD-OCT system’s image visibility close to the edges of the ranging depth was comparable to that obtained with SS-OCT. The blue arrows in Fig. 6(b) highlight the visible but minor artifacts created from the merging process.

## 4 Discussion

In this paper, we have introduced a dual-interferometer approach for extending the effective imaging range of conventional SD-OCT systems. A combination of the images corresponding to the two interferometers using the algorithm described in Sec. 2 resulted in a sensitivity enhancement of over 14 dB at the 6-mm depth as compared to single interferometer, conventional SD-OCT systems. This interferometer configuration and algorithm resulted in a sensitivity roll-off of ∼14  dB/6  mm making our system suitable for *in vivo* endoscopic OCT imaging of large luminal organs such as the small intestine and colon. We validated our system in swine colon *ex vivo* and swine duodenum *in vivo*, demonstrating an imaging range enhancement and comparable ranging depth performance to that of conventional SS-OCT systems.

This proposed SD-OCT imaging-range augmentation approach has advantages over previously reported dual-reference-arm techniques.^17^ First, the proposed system is motion insensitive; the fixed delay line (∼1.5-km single mode fiber) at the output of the second interferometer ensures that the A-lines arriving serially at the camera from the two interferometers are acquired from the same spatial position within the same temporal window, hence eliminating any possibility for artifacts caused by sample motion between successive A-lines.^17^ The fixed delay line also suppresses inter-interferometer interference artifacts between the two interferometers arising from limited isolation ratio (∼20  dB) of the OS, by shifting the signal from the second interferometer out of the coherence window of the first interferometer before detection. Second, to resolve the complex conjugate ambiguity that results in aliasing artifacts in images corresponding to the second reference arm, Rollins et. al.^17^ used a B-scan Doppler shift method that required dense transverse sampling. This dense transverse sampling may potentially limit scan range and speed and is not required with our proposed method for extending the imaging range.

When compared to SS-OCT, the sensitivity of both SS and SD-OCT systems fall by half (or 6 dB) at a depth $z_{6\;\mathrm{dB}}$ proportional to $\lambda_{0}^{2}/\delta \lambda$, where $\lambda_{0}$ is the center wavelength of the optical source and δλ is the sampled spectral resolution.^26^ The depth $z_{6\;\mathrm{dB}}$, half of the instantaneous coherence length of a SS optical source, is determined by the instantaneous linewidth of a SS optical source and the spectrometer’s spectral resolution in SS and SD-OCT systems, respectively. Today’s SS-OCT systems inherently have a superior ranging depth (sensitivity roll-off: ∼6  dB/4  mm) compared to SD-OCT systems (sensitivity roll-off: ∼20  dB/4  mm) owing to their large coherence lengths.^27^^,^^28^ The major but practical contributing factor to increased sensitivity roll-off in SD-OCT systems is the finite camera pixel size, which combined with inter-pixel cross-talk diminishes fringe visibility of higher spatial frequencies. However, SD-OCT can be conducted for wavelengths where semiconductor gain media required for swept sources do not exist, allowing very broad bandwidth imaging with the highest axial resolutions since the axial resolution is increased with the bandwidth of the optical source used.^29^^,^^30^ SD-OCT also offers better phase stability due simultaneous acquisition of A-scan sample points as opposed to SS-OCT where sample points are acquired serially. These advantages of SD-OCT make solutions such as ours to increase SNR at the edges of a 6 mm ranging depth useful for certain medical applications such as imaging of large luminal organs, e.g., the esophagus, small intestine, terminal ileum, and colon, to screen for conditions such as Barrett’s esophagus, celiac disease, and Crohns disease. The merging of the two images introduces minor artifacts deeper in the merged images away from the surface. This limitation is likely not to be significant as valuable tissue OCT information usually lies closer to the probe’s surface. The proposed system also suffers from the main drawback of frame rate loss by a factor of two since twice as many A-lines are required to create one final frame. Nonetheless, at an effective A-line rate of 70 kHz, this 1310-nm SD-OCT fares reasonably well in comparison to 1310-nm SS-OCT systems that are in clinical use. While the current real-time display frame rate of the merging scheme of 12 fps is acceptable for most applications, further optimization of the C++ implementation (increasing the number of threads) or adoption of GPU real-time processing should be able to yield the full 27-fps real-time display.

## 5 Conclusion

We have developed a dual interferometer SD-OCT system that uses an OS and a ∼1.5-km fiber-optic delay line to acquire OCT images using a single spectrometer. The dual interferometer architecture facilitates OCT imaging sensitivity roll-off augmentation to within 14 dB for ∼6-mm imaging range. The system further ensures sequential acquisition of interference signal from the two interferometers originating from the same location of the sample, mitigating motion artifacts that may occur between successive A-lines. The system is compatible with catheter-based endoscopic helical scanning with a >14-dB enhancement in sensitivity for the sample located at ∼6-mm optical delay.
